# Periodical Headache

**Published:** 1872-01

**Authors:** F. Bradnack


### ART. IV.—*Periodical Headache. A New Method of Curative Treatment.* BY F. BRADNACK, M. D.

Inasmuch as even so high an authority as Professor Austin Flint, declares it necessary to place the affection known as "periodical headache" in the (medically speaking,) unpleasing category denominated *opprobia medicorum,* no apology will be needed from any laborer in the ranks of the profession, who can demonstrate his ability to throw even one ray of light upon the therapeutics of this most distressing malady. And, inasmuch as persistent and perpetual progress is one of the proudest boasts of our blessed art, I shall venture, without any further preface, to detail some of my experience in the treatment of this affection, in the hope of demonstrating the truth of the proposition that periodical headache can now be generally, if not invariably, driven from its hitherto safe retreat in the *opprobia medicorum,* and compelled, at least, in most instances, to succumb to the scientific application of improved therapeutics.

In Dr. Flint's work on "The Principles and Practice of Medicine," under the head of *Cephalagia,* page 694, are the following words: "Periodical headache, as regards successful treatment, belongs among the opprobia of the medical art; yet, in not a few cases, much benefit may be derived from treatment." In quoting from Dr. Flint the above sentence, in connection with a proposed improved method of therapeutics in this malady; it seems to be a duty, while it is, at the same time, a pleasure to remark that, than myself, no one can hold in higher estimation Dr. Flint's professional abilities, or entertain for his opinions a greater respect.

I believe that one prime reason why we have hitherto been unsuccessful in the treatment of periodical headache, relates to diagnosis; for my experience in a number of cases leads me to infer that the pathological condition in this malady is, if not always, *almost* always the same. At the risk of creating some surprise, I shall venture the assertion, (believing that future accurate observation will verify it,) that this pathological condition is *congestion*; sometimes active, that is, arterial, and, sometimes passive, or venous congestion; though, probably, in most cases, a combination of both these forms; the arterial form usually predominating. In other words, the trouble is mechanical; the pain and distress being the natural result of pressure upon the brain substance, occasioned by abnormal distension of the cerebral blood-vessels.

That this pathological condition in the eight or nine cases of periodical headache I have recently treated, has not been conjectural, the following facts will prove. In each case there have been, more or less marked, the following signs and symptoms: 1. Throbbing of the carotid and temporal arteries; 2. Congestion of the eyes, in some cases occasioning coryza; 3. A sensation on the part of the patient of intense intracranial tension, described popularly by saying that the head "feels as if it would burst"; 4. Redness of part of, or of the entire face; 5. A partial loss of the power of co-ordinating muscular movements, occasioned, I conjecture, by extreme vascular pressure upon the cerebellum; 6. Occasional vomiting; 7. *tinnitus aurium,* often very violent. These are the symptoms and signs I find invariably present; and on which I base the assertion that cerebral conjestion is the almost invariable cause of these headaches. From these it will be seen that the diagnosis is, so far, not based on theory, but on facts, which, as we all know, are stubborn things. But, aside from these positive signs and symptoms, I find many others present in greater or less degree, some of which go, either negatively or indirectly to substantiate the position advanced. Among these are: 1. Anorexia; 2. Muscæ volitantes, occasioned doubtless by congestion of the retinæ; 3. Extreme coldness of the extremities; 4. Often a decrease of heat, as indicated by the thermometer, of large portions of the cutaneous surface, indicating a retreat of the blood inwards *somewhere*; 4. Numbness and partial temporary paralysis of the upper extremities, caused, unquestionably, by intracranial pressure; 5. Difficult respiration, amounting in some instances to dyspnœa. This latter symptom, taken in connection with the fact of the existence of abnormal heat in the upper portion of the cervical spine, and lower external regions of the occipital bones, appear to me to indicate, in common with the adjacent and superjacent congestion, an abnormal pressure on the medulla oblongata, implicating the origins of the pneumogastric and spinal accessory nerves.

If, in many other diseases, we were in possession of half as many positive and negative symptoms, should we not be confident of our diagnosis? Believing that an affirmative answer will be generally accorded to this question, we next pass to briefly consider the question of the causation of the malady under discussion. To arrive at positive conclusions on this head appears to me to be by no means so easy a task as in the case of the diagnosis. Yet in many cases, the probable causative influences may be elucidated with much certainty. They may be divided into predisposing and exciting causes. In regard to the predisposing causes, that hereditary influences have frequently considerable to do with the production of this disease, would seem to be probable, both theoretically, and upon clinical evidence. We know the power of hereditary influences in other neurotic affections; for instance, epilepsy and hysteria. We also know that the existence of one specific nervous disease in the parent or parents, frequently seems to prepare the way for, or to eventuate in, a different nervous disease in the child. Indeed, it is not reasonable to suppose that any nervous lesion, or any persistent abnormal condition of the general nervous system of a parent, (for instance, chronic alcholism, or progressive locomotor ataxy,) will be quite likely to have some influence upon the offspring. This is not asserting that the children of parents affected with epilepsy, must necessarily be epileptics; or, that the existence of a specific nervous lesion in a parent necessarily results in the production of the same specific disease in the child, but merely, that the existence of serious permanent nervous lesions in the parent, tend in some manner to influence the nervous system of the offspring; perhaps only to the extent of rendering it more vulnerable to disease. To allow the possibility of the latter effect, is amply sufficient for the argument. Next, the exciting causes. Given the predisposition strongly marked, it is hardly an exaggeration to say that almost anything may act as an exciting cause of these headaches. The exciting causes are, to use a popular expression, almost too numerous to mention. Prominent among them, however, in my existence are the following: Mental emotions; excessive brain work; ingestion of improper food, or, of too large a quantity of proper food; cold; heat; constipation, (especially a loaded condition of the rectum;) abuse of stimulants, as alcohol and tobacco; the improper use of opium; and so on. In the female they may be amenorrhœ, dysmenorrhea, uterine or ovarian irritation; uterine displacements, especially retroflexion or retroversion of the uterus, by reason of their pressure on the rectum, thereby causing obstipation, or inducing constipation of the bowels.

I have observed, that in every case of periodical headache I have treated, or with the history of which I have become acquainted, without one exception; constipation, often chronic prevailed, which leads me to remark, that in some unexplained manner, the pressure exerted upon the rectal nerves by a fecal cylinder, appears invariably to aggravate these headaches, and doubtless, frequently acts as a causative influence.

Inasmuch as one of the specific effects of alcohol is to induce cerebral congestion, it is manifest that an excessive use of this article must, of necessity tend to excite the affection in question. That it does so excite it, is, I believe, provable by irrefragable evidence. This remark will, of course, apply with increased force to the use of opium. That the phenomena of cerebral congestion and anœmia are regulated by the vaso-motor nerves, recent histological researches appear to have demonstrated. If this be really so, we have here a key to many mysteries; for, if it be, as it undoubtedly is, a fact that certain drugs have the power to affect these nerves, and thereby either to dilate or constringe the arteries to which they are distributed, we find ourselves furnished with indications of the highest importance as to treatment, if not with a perfect therapeutical key.

But, after we have enumerated all the known exciting causes of this disease, we shall often find ourselves quite in the dark, both as to the pathology and treatment, if we fail to allow for, in at least many instances, an *occult, subtle and inexplicable constitutional*

*tendency* to this malady, which can no more be explained or accounted for, than can the phenomenon of menstruation, or the existence of individual idiosyncrasies. Time spent in an endeavor to discover and elucidate the nature of this tendency, would probably prove to be as effectually wasted, as if spent in the mathematical endeavor to trisect an angle. It is sufficient for practical purposes to take this tendency into account, and to allow for it.

It will be seen from Dr. Flint's above quoted statement, that the prognosis of periodical headache, has hitherto been exceedingly unfavorable as regards any method of permanent relief; which remark brings us to the question of treatment.

Having, I trust, as hearty a disbelief in, and dislike of most so-called specific plans of treatment, (believing it to be usually quite impossible to lay down any specific or invariable treatment for disease,) I nevertheless venture the statement, that, for periodical congestive cephalalgia we may employ a method of treatment, which, if not specific aproximates, so nearly to a specific character, as to, in many instances, honestly deserve the appellation. I am aware this is a strong statement, but I am, (I trust not unreasonably) so confident of its truth, as to be anxious to subject it to the *experimentum crucis* of actual test, in the faith that when weighed in scientific balances, it will not be found wanting.

The treatment of this malady, divides itself naturally into two parts; first, that proper during the attack; second, that appropriate in the internal. I believe we possess remedies capable, in most instances, of entirely, or nearly entirely, allaying the pain during the attack; and others administered during the intervals of effecting in a few months a permanent cure. To say that I have wholly originated this method would be to make a strong claim; but, that it has never in this disease been systematically employed before, I am certain.

In the proposed treatment of this disease, we, of course, adhere to general principles. If there exist complications, whether or no they assumed to act as exciting causes, they should be removed, or so far as possible, paliated. If there be constipation, the usual treatment for constipation is indicated. If examination reveals the existence of a uterine displacement, it should be remedied. If tobaccos are used in excess, they must be either discontinued or used in moderation. Suppose the treatment to be commenced the day after an attack of headache. Assuming the non-existence of any important physical lesion, I find it advantageous, provided there are no contraindications, to begin by the administration at night, one or two of the following pills, which, during the entire course of treatment (say six months) may be given once in three weeks:

℞ Mass hyd.
Ext. coloc. com.
Pulv. aloes soc. a a, ℈ j.
Pulv. ipecac. gr. vj.
M. Ft. pil., no. xij.

This pill to be followed in the morning by one (1) drachm of sulphate of magnesia.

As a permanent medicine, I then prescribe three (3) drops of liquor potassæ arsenitis, to be taken in one (1) drachm of water after each meal, for certainly three, and usually six months its use being suspended one day every three weeks when the above pill is taken.

If the patient be delicate, and complains much of coldness of the extremities during the attacks, and frequent chilliness during the intervals, the following prescription is substituted for the liquor potassæ arsenitis:

℞ Liq. arsenicalis hydrochlorici, ℥ ss.

Quiniæ disulphat. gr. xij.

Liq. ferri perchloridi, ʒ ij.

M. Aquæ, f ℥ vj.

(*S.* One tablespoonful in a wine-glassful of water, twice a day, after meals.)

When an attack of headache begins, I adopt the following plan, with minor modifications according to existing circumstances and complications. I direct the patient to sit in an easy chair, (avoiding the recumbent position, as tending to cerebral congestion by means of gravitation,) and to place his, or her feet in a hot bath of mustardized water, the hands also in a similar hot bath, minus the mustard; and if it can be tolerated, (though females frequently can not tolerate it,) a bag of pounded ice to be placed upon the head, covering as much as possible of the occipital region, and thereby bringing a decongestive influence to bear upon the cerebellum and the medulla oblongata. These accessory measures to be followed by a dose of the following medicine ;

℞ Potassii bromid. ʒ vj.

Ammon. bromid. ʒ ij.

Potassii iodidi, gr. vj.

M. Infus columbæ, f ℥ iiij.

*S.* One dessert-spoonful in an ounce of water.

One or two doses of this prescription will usually suffice either to very greatly palliate, or else entirely relieve the most distressing and agonizing headache, provided only it belongs to the class under consideration. But, to produce its best effects, this remedy should be administered as early as possible after the commencement of the attack. In some cases patients experience prodromic symptoms. When these occur, the threatened attack may often be rendered abortive by the timely administration of the medicine.

As it has been frequently demonstrated by Professor W. A. Hammond, of New York, that the bromide of potassium possesses the power of decreasing the amount of blood in the skull, I, of course, claim no originality in using that remedy in this connection, in which the chief pathological condition is cerebral congestion. But in combining with this drug the bromide of ammonium, and a small amount of iodide of potassium, and in exhibiting the combination in a little infusion, (the infusion of colombo,) I claim originality. That the above formula constitutes altogether the most eligible manner of combining these articles into a weapon of offense against the disease, I feel convinced by repeated trials of it.

In this plan of treatment, the prescription containing the bromides is given just before, or during an attack, with the object of reducing the cerebral congestion, and thereby allaying the paroxysm of headache. That it is capable of doing this, I am satisfied by ocular evidence, as well as by asservations of patients. I am at the present time treating a lady who reports herself as invariably relieved of an attack of headache by the use of the above prescription alone, after having suffered these attacks for eleven years, and being treated by numerous physicians, without receiving a particle of benefit.

The prescription containing arsenic, or the simple liquor potassæ

arsenitis, is given with the object of changing, or overcoming the subtle constitutional tendency to these headaches, previously alluded to. In my hands it produces this affect, whether it do so through the medium of the vaso-motor nerves, or by a general tonic health-producing impression on the entire nervous system, or upon the sympathetic nerve, I am, of course, unable to say; but, that it effects after a few months a permanent cure of the disease, I am able to assert, in at least a certain number of instances, sufficient to induce me to believe, that if scientifically administered, and faithfully persevered in for six or seven months, it will in the vast majority of cases prove successful in the hands of the scientific practitioner.

Without undertaking to explain the exact action of arsenic, I cannot but believe from my observations of its action in this disease that it must exert a decongestive influence upon the entire encephalon. I would venture the conjecture that it may exert *permanently* the same decongestive action upon the brain, and upper portion of the spinal marrow, that the bromide of potassium exerts temporarily. If this be so, we have a sufficient explanation of its efficacy in the disease under consideration.

In conclusion, if it shall be possible by what has here been said to contribute even an iota towards the relief of this distressing, and hitherto nearly incurable malady, the knowledge of having so contributed will constitute for the writer a most ample reward.

